# Enzyme Immobilisation on Amino-Functionalised Multi-Walled Carbon Nanotubes: Structural and Biocatalytic Characterisation

**DOI:** 10.1371/journal.pone.0073642

**Published:** 2013-09-12

**Authors:** Madan L. Verma, Minoo Naebe, Colin J. Barrow, Munish Puri

**Affiliations:** 1 Centre for Chemistry and Biotechnology, Geelong Technology Precinct, Deakin University, Waurn Ponds, Geelong, Victoria, Australia; 2 Institute for Frontier Materials, Geelong Technology Precinct, Deakin University, Waurn Ponds, Geelong, Victoria, Australia; Russian Academy of Sciences, Institute for Biological Instrumentation, Russian Federation

## Abstract

**Background:**

The aim of this work is to investigate the structure and function of enzymes immobilised on nanomaterials. This work will allow better understanding of enzyme-nanomaterial interactions, as well as designing functional protein-nanomaterial conjugates.

**Methodology/Principal Findings:**

Multiwalled carbon nanotubes (MWNTs) were functionalised with amino groups to improve solubility and biocompatibility. The pristine and functionalised forms of MWNTs were characterised with Fourier-transform infrared spectroscopy. Thermogravimetric analysis was done to examine the degree of the functionalisation process. An immobilised biocatalyst was prepared on functionalised nanomaterial by covalent binding. *Thermomyces lanuginosus* lipase was used as a model enzyme. The structural change of the immobilised and free lipases were characterised with transmission electron Microscopy, X-ray photoelectron spectroscopy, Fourier-transform infrared spectroscopy and Circular dichroism spectroscopy. Biochemical characterisation of immobilised enzyme showed broader pH and thermal optima compared to soluble form. Reusability of the immobilised enzyme for hydrolysis of long chain esters was demonstrated up to ten cycles.

**Conclusion/Significance:**

Lipase immobilised on MWNTs has exhibited significantly improved thermal stability. The exploration of advanced nanomaterial for enzyme immobilisation support using sophisticated techniques makes nanobiocatalyst of potential interest for biosensor applications.

## Introduction

Various nanostructured forms such as nanoparticles, nanofibres, nanotubes, and nanocomposites have been employed as novel supports for enzyme immobilisation and stabilisation [1]–[4]. These robust nanoscaffolds have an inherently large surface area which leads to high enzyme loading and consequently high volumetric enzyme activity. Amongst various nanomaterial forms, carbon nanotubes possess unique structural, mechanical, thermal and biocompatibility properties. Carbon nanotubes have attracted considerable research interest for potential biotechnological applications, particularly in biosensors development [5]–[7]. The efficiency of nanomaterial can further be improved by the process of surface functionalization [8]. The surface functionalisation of nanomaterials involves grafting of desirable functional groups onto their surface to obtain nanomaterials with desired properties [9]. This functionalisation can affect their dispersability and interactions with enzymes, thus significantly altering the catalytic activity of the immobilised enzyme [10]. Although numerous attempts have been made to immobilise enzymes with nanomaterials, studies on the impact of nanomaterial properties (such as composition, morphology, and surface chemistry) on the structure and function of conjugated enzymes are hitherto sparse [5]. Investigating the structure and function of enzymes immobilised on nanomaterials will be crucial for developing a better understanding of enzyme-nanomaterial interactions, as well as for designing functional protein-nanomaterial conjugates [11].

Enzymes in the free form are fragile and prone to denaturation in extreme conditions of pH, temperature and organic solvents. To improve their stability, enzymes have generally been immobilised on a solid carrier [12]. Enzyme immobilisation can facilitate enhancement of catalytic stability, selectivity and reusability of the enzymes [13], [14]. Different techniques of enzyme immobilisation have employed either physical or covalent methods. Adsorption is a relatively simple method as it is a chemical free enzyme binding process [15]. However, leaching of the enzyme from the immobilised enzyme preparation after a certain number of reuses has limited its application at commercial scale. The aforementioned shortcoming can be overcome by the use of a covalent-binding using suitable cross linkers such as glutaraldehyde [13], [16]–[18]. Nanomaterial activation of either glutaraldehyde or carbodiimide has been most commonly and successfully employed for covalent immobilisation [3], [19]. Covalent binding methods produce relatively stable immobilised enzyme preparations with more reusability as compared to the physical adsorption method [2], [18]. Recently, researchers have immobilised lipase on carbon nanotube through adsorption for ester synthesis in non-aqueous medium [10], [19], [20]–[22], however, associated structural changes in the immobilised enzyme are not documented in the literature.

In this present work, multi-walled carbon nanotubes (MWNTs) were amino-functionalised with ethylenediamine and examined for the degree of functionalisation. *Thermomyces lanuginosus* lipase was then covalently immobilised onto the functionalised MWNTs supports using a glutaraldehyde cross-linker. Enzyme post immobilisation structural changes were analysed using various spectroscopy techniques. The free and MWNTs bound lipase was biochemically characterised with respect to ester hydrolysis in aqueous medium. The catalytic efficiency of the immobilised lipase, in terms of thermal stability and reusability, was also studied.

## Materials and Methods

### Materials

Multi-walled carbon nanotubes (MWNTs; length of 0.5-2 micron and outer diameter of 20–30 nm) were purchased from Arry International. All other chemicals used to functionalise MWNTs were purchased from Sigma-Aldrich. Lipase from *Thermomyces lanuginosus* (EC 3.1.1.3; 100,000 U/g; protein = 22.4 mg/mL), a purified enzyme in a liquid preparation, was obtained from Sigma-Aldrich Chemical Co., Australia. A protein assay kit (Bio-Rad protein dye reagent concentrate) and Tris-buffer were procured from Bio-Rad. p-Nitrophenyl palmitate, p-Nitrophenol, Triton-X-100, Phosphoric acid and Gum acacia, were purchased from Sigma-Aldrich Chemical Co., Australia. Deionised double-distilled water with 18.2 megaohm cm^−1^resistivity was used in making solutions. All chemicals (analytical grade) were used as received without any further purification.

### Functionalisation and Characterisation of MWNTs

The as-received MWNTs were first treated with a 3∶1 v/v mixture of concentrated H_2_SO_4_ and HNO_3_ in a round-bottomed flask, and refluxed at 120°C for 30 min. After cooling to room temperature, the mixture was vacuum-filtered on a 0.2 mm pore Polytetrafluoroethylene (PTFE) membrane and washed with distilled water until no acid residual was present. The oxidised nanotubes were then redispersed and washed in a NaOH solution (0.01 M) followed by washing with distilled water until pH neutral. Finally, the product was redispersed in HCl solution (0.01 M), and repeatedly washed with distilled water until pH of the filtrate was neutral. The resulting oxidised nanotubes (100 mg) were dried in a vacuum oven at 80°C for 8 h and subsequently dispersed in ethylenediamine (EDA, 60 mL) to obtain amino-functionalised carbon nanotubes. The coupling agent, HATU (O-(7-Azabenzotriazol-1-yl)-N,N,N′,N′-tetramethyluroniumhexafluorophosphate, 8 mg) was added to increase the reaction yield and the dispersion was sonicated for 4 h at 40°C. The obtained product was diluted with 300 mL of methanol and vacuum-filtered using a 0.2 mm pore PTFE membrane filter, after which the filtrate was washed extensively with excess methanol following by vacuum drying at 60°C for 8 h. Thermogravimetric analysis (TGA) for pristine MWNTs and amino-functionalised MWNTs was conducted under a nitrogen atmosphere using a Perkin Elmer at a heating rate of 10°C/min.

The FTIR analysis of functionalised nanotubes was carried out to confirm the presence of amino groups in the functionalised carbon nanotubes. ATR-FTIR measurements were conducted using an Alpha FTIR spectrometer (BrukerOptik GmbH, Ettlingen, Germany) equipped with a deuterated triglycine sulfate (DTGS) detector and a single-reflection diamond ATR sampling module (Platinum ATR QuickSnap™). The ATR-FTIR spectra were acquired at 4-cm^−1^ spectral resolution with 64 co-added scans. Blackman-Harris 3-Term apodization, Power-Spectrum phase correction, and zero-filling factor of 2 were set as default acquisition parameters using the OPUS 6.0 software suite (Bruker). Background spectra of a clean ATR surface were acquired prior to each sample measurement using the same acquisition parameters.

The size and morphology of the amino-functionalised and enzyme conjugated nanotubes were characterised by transmission electron microscopy (TEM) using a JEOL 2100 M microscope (Frenchs forest, NSW, Australia) with an electron beam energy of 200 kV. TEM specimens were prepared by evaporating a drop of aqueous nanotube dispersion on the carbon-coated specimen grid. Once evaporated, the sample was loaded into the microscope for imaging.

### Covalent Immobilisation of Enzyme on Functionalised MWNT

Lipase was immobilised on the functionalised nanotubes (Fig. S1). The nanotubes (5 mg/mL) were first sonicated in the aqueous buffer (50 mM, Tris Buffer, pH 9.0) for 30 min to ensure homogenously dispersion. Surface-activation of the amino-functionalised nanotubes was achieved by treating the nanomaterials with 1 M glutaraldehyde solution [13]. Support activation was carried out at 25°C in a shaker (250 rpm) for 1 h. The activated support (5 mg/mL) was removed by centrifugation (7000 rpm, 20 min), washed at least five times with 20 mL of distilled water to remove excess glutaraldehyde, and then washed with washed with assay buffer. Lipase was immobilised onto functionalised nanotubes through covalent linkage between amino group of nanotube and amino group of enzyme through a glutaraldehyde cross-linker. The purified enzyme (5–20 mg in 50 mM Tris buffer, pH 9.0) was mixed with the activated support (5 mg/mL) in an enzyme assay buffer to optimise the nanotube and protein-loading ratio. The immobilisation process was performed at 25°C in a shaker (150 rpm) for 10 h. Non-covalently adsorbed protein was removed thereafter by thorough washings of the nanotubes with saline solution (1 M NaCl) followed by enzyme assay buffer until no hydrolytic activity was detected in the washings. The supernatant was used for protein analysis. The washed carrier was directly used for the determination of enzyme activity and thermal stability.

The amount of immobilised enzyme was calculated by measuring the proportion of the enzyme remaining in the supernatant of the enzyme assay buffer after the immobilisation process. The amount of protein in the supernatant at each washing cycle was added to calculate the total amount of free enzyme until no protein was leached. It was found that the quantity of immobilised lipase was 0.50 mg protein/mg MWNT at equal ratio of protein and functionalised nanotube in mg was achieved using this immobilisation method. Activity retention of the immobilised form was 65% than the free enzyme. Protein content of the enzyme solution, before and after immobilisation, in the washing buffer was determined by the Bradford method with Bio-Rad protein dye reagent concentrate and bovine serum albumin as a standard protein [23].

### Structural Characterisation of Immobilised Lipase

The enzyme-immobilised nanotubes were characterised by transmission electron microscopy (TEM). The size and morphology of nanotubes was observed by TEM. The sample for TEM analysis was obtained by placing a drop of well-dispersed nanotubes solution onto a carbon grid coated, followed by drying the sample at ambient temperature.

X-ray photoelectron spectroscopy (XPS) was used to investigate the chemical composition of the pristine, amino-functionalised and enzyme immobilised carbon nanotubes. The spectra were acquired using a Vacuum Generators Escalab V unit equipped with a non-monochromatic A1 Ka source at 200 W (20 mA at 10 kV) and a hemispherical analyser operating in the fixed analyser transmission mode. The photoemission peak areas of each element was normalised by the sensitivity factors of each element tabulated for the spectrophotometer used.

Circular dichroism (CD) spectroscopy was used to monitor the secondary structure of the native lipase and MWNT immobilised lipase. The UV CD spectra (200–260 nm) were recorded on a JASCO J-810 CD instrument (JASCO) with a bandwidth of 0.5 nm and a scan speed of 50 nm/min. Cell length was 10 mm. In all measurements, the lipase concentration was kept at 50 µg/mL. All CD measurements were performed at 25°C in diluted Tris buffer (10 mM, pH adjusted with phosphoric acid to 9.0). The CD spectrum of amino-functionalised MWNT was similarly recorded. All the spectra were corrected by subtracting a blank spectrum (without lipase). Each scan was repeated five times, after which the spectra were averaged.

### Determination of Enzyme Activity

The activity of free and immobilised enzyme was measured by a colorimetric method [24]. The reaction mixture contained 80 µL of p-nitrophenyl palmitate (3 mg/mL; p-NP palmitate prepared in isopropyl alcohol), the test sample (lipase), and Tris buffer (0.05 251658240M, pH 9.0) containing 0.4% (w/v) Triton-X-100 and 0.1% (w/v) gum acacia (2.9 mL), to make final volume of 3 251658240mL. The reaction mixture was incubated at 60°C for 10 min in a water bath. The reaction was terminated by holding the reaction mixture at −20°C for 10 251658240min. An appropriate control with a heat-inactivated enzyme (5 min in a boiling-water bath) was included with each assay. The liberated 4-nitrophenol was measured by UV-Vis Spectrophotometer (Shimadzu, Japan) at 410 nm and its concentration calculated from a plot constructed for standard p-nitrophenol. One unit of enzyme activity is defined as 1 µmol of p-nitrophenol liberated per minute under standard assay conditions (pH 9.0, 60°C). Activity of immobilised lipase (20 µg) was measured with p-NP palmitate (equal amounts corresponding to free enzyme) at 60°C. All additives, including the buffer, were pre incubated for a short period (5 min) before the enzyme was added to initiate the reaction. All enzyme assays were performed in triplicate and reported as mean values ±SD.

### Biochemical Characterisation of the Free and Immobilised Lipase

The optimum pH for immobilised and free lipase activity was determined using standard assay conditions in Tris buffer at varying pH (pH 7.0–9.5) at 60°C. Relative activities of the free and immobilised lipases at selected range of pH values were quantified relative to the control activities, and expressed as fractions thereof. These control activities, those representing 100% relative activity, were taken to be at pH 9.0 for both the free and immobilised lipases.

The effect of temperature on free and immobilised lipase activity at pH 9.0 was determined by varying the temperature in the 30–80°C range. Other experimental conditions adhered to the standard assay protocol. The activity at 60°C was taken as control for the calculation of 100% relative activity for the free and immobilised lipase.

### Determination of Kinetic Parameters

Kinetic parameters of free and immobilised lipase at 60°C were determined using different p-NP palmitate concentrations in the range of 10–60 mM in 50 mM Tris buffer (pH 9.0) respectively. *K_M_* and *V*_max_ values of free and immobilised lipases were calculated via non-linear regression fitting of the of the Michaelis-Menten equation using Prism 6 (Graphpad Software, Inc., CA, USA).

### Thermal Stability of Free and Immobilised Enzymes

Thermal stability of the free lipase was quantified in terms of the loss in enzyme activity when incubated at selected temperatures (60, 70 and 80°C) in the absence of a substrate. The activity of free lipase at pH 9.0 (Tris buffer, 50 mM) was observed at regular intervals between 30 and150 min. Similarly, to determine the thermostability of the immobilised enzyme, the immobilised lipase was incubated at the above mentioned selected temperatures in the absence of a substrate. After varying periods of time, the residual lipase activity was determined. The relative activity of the free and immobilised lipase without incubation was defined as control and arbitrarily attributed 100% relative activity for each of their respective reactions.

### Biocatalyst Reusability Study

The reusability of the immobilised preparation was assessed at 60°C by carrying out the hydrolysis of a long chain ester (p-NP palmitate) under standard assay conditions. After each cycle, the immobilised enzyme was removed by centrifugation (7000 rpm, 20 min). The immobilised lipase was collected and washed simultaneously with deionised water and enzyme assay buffer. In running the second cycle, the immobilised enzyme was resuspended in fresh enzyme assay buffer and added to fresh p-NP palmitate. The activity of the immobilised enzyme after the first cycle was defined as the control and attributed a relative activity of 100%. Each cycle is defined here as the complete hydrolysis of the substrate present in a reaction mixture.

## Results and Discussion

The development of an efficient functionalised nanotubes support for enzyme immobilisation, using a covalent binding method for investigating its influence on the biocatalyst structure-function relationships, was the primary objective of the work. Analytical techniques have been used to characterise *in situ* structural stability of enzymes immobilised on supports [25]. The conformational changes of enzymes upon immobilisation and confirmation of the success of the immobilisation methods have been investigated using sophisticated analytical techniques such as TEM and spectroscopy (CD, FTIR and XPS).

**Figure 1:**
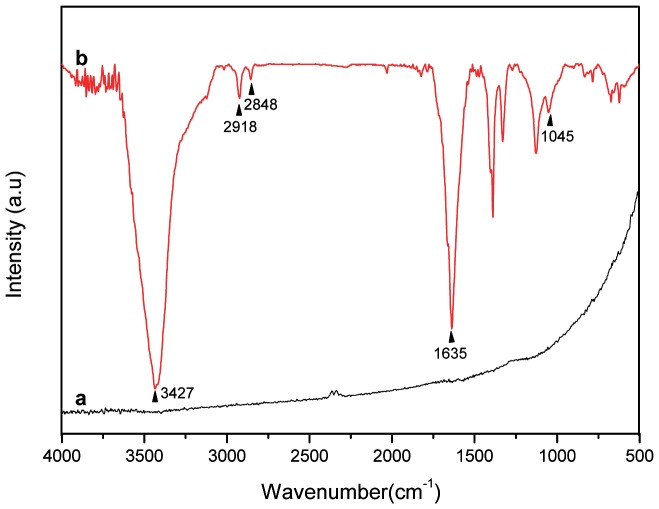
FTIR spectrum of (a) pristine MWNT and (b) amino-functionalised MWNT. Footnote: MWNT treated with a mixture of concentrated H_2_SO_4_ and HNO_3_ (v/v 3∶1), and refluxed at 120°C. After cooling to room temperature, the mixture was vacuum-filtered on a 0.2 mm pore Polytetrafluoroethylene membrane. The oxidised nanotube was washed in a NaOH solution. Finally, the product was redispersed in HCl solution. The obtained oxidised nanotubes were dried in a vacuum oven and then were dispersed in ethylenediamine to obtain amino-functionalised MWNTs. The coupling agent,O-(7-Azabenzotriazol-1-yl)-N,N,N′,N′-tetramethyluroniumhexafluorophosphate was added to increase the yield of reaction and the dispersion was sonicated. The product was diluted with methanol and vacuum-filtered using a 0.2 mm pore PTFE membrane filter, after which the filtrate was washed extensively with methanol following by vacuum drying.

FTIR spectra for pristine MWNT and amino-functionalised MWNT are shown in Figure 1. Since the pristine carbon nanotube is not sensitive to FTIR, no obvious peak is observed. This is similar to previous FTIR findings [26]. The IR spectrum of the amino-functionalised MWNT (MWNT-CO-NH (CH_2_)_2_-NH_2_) is shown in Figure 1. The absorption band at 1635 cm^−1^ is assigned to the stretching vibration mode of C = O in the amide group. The NH_2_ stretch band appeared at 3427 cm^−1^, whereas the peak at 1045 cm^−1^ is attributed to the C-N bond stretch vibrations. Bands at 2918 and 2848 cm^−1^ represent the stretching of the CH_2_ group [27].

**Figure 2:**
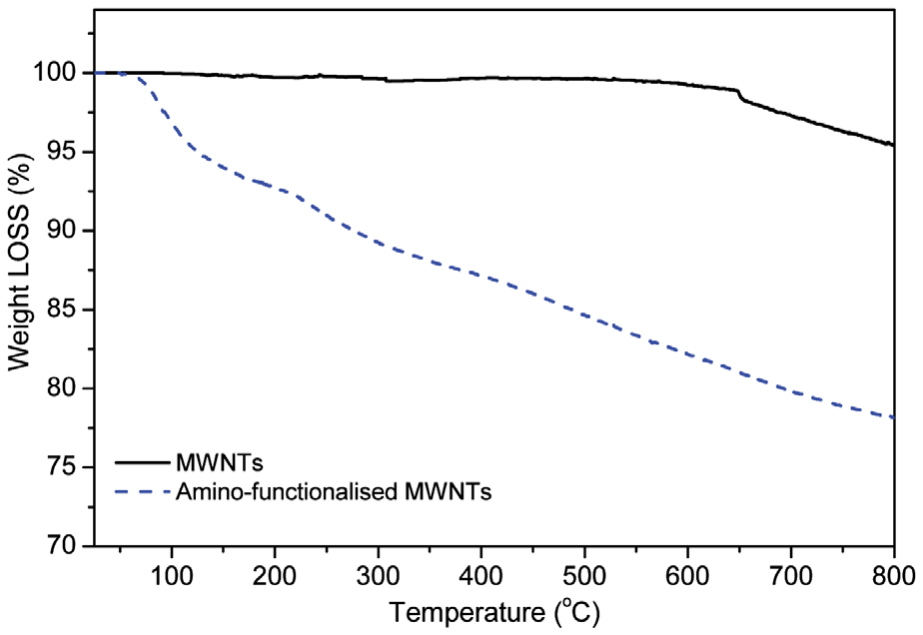
Thermal gravimetric analysis (TGA) plot for pristine MWNT and amino-functionalised MWNT.

**Figure 3:**
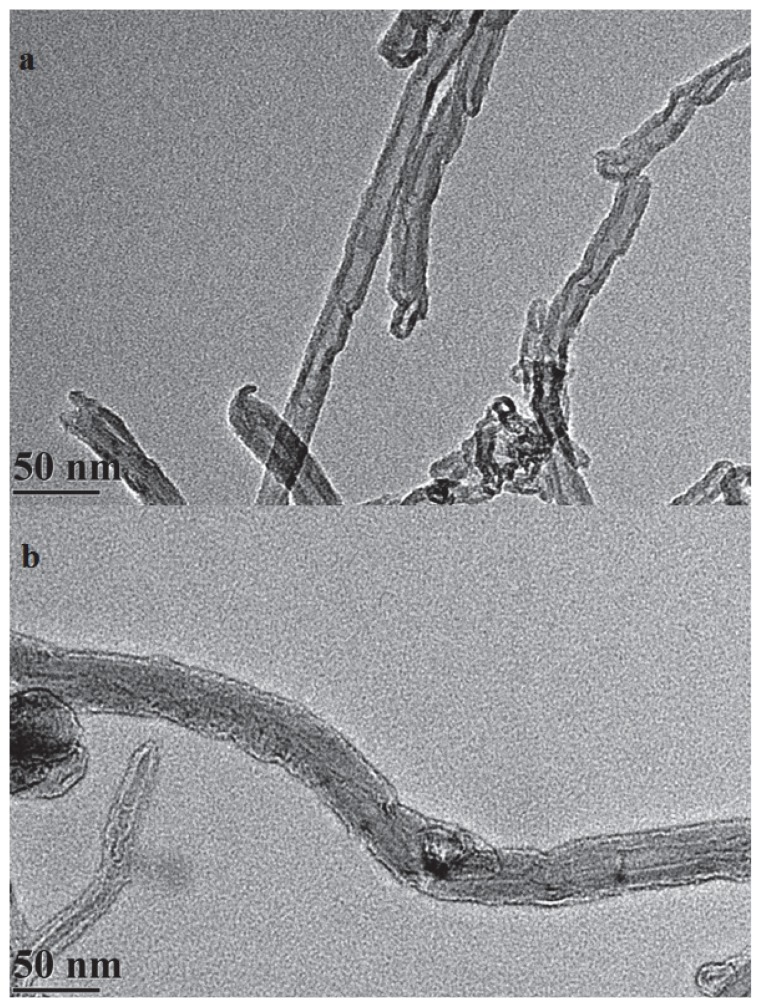
TEM images of functionalised MWNT (a) and MWNT bound lipase (b).

The degree of functionalisation can be determined using Thermal gravimetric analysis (TGA). Figure 2 shows the TGA results for pristine MWNT and amino-functionalised MWNT. There is 4.5% weight loss for pristine MWNT which could be due to the removal of oxidised carbon groups. For amino-functionalised MWNT, the weight loss is about 22% which is attributed to the presence of amino groups on side walls of MWNT. Therefore the degree of functionalisation estimated to be 1 added in every 41 carbons, which is higher than that of amino functionalisation of nanotube through diazotiazation [26]. Identically processed functionalised MWNT with and without the immobilised enzyme were viewed by electron microscopy (Fig. 3). The size and morphology of the nanomaterial was observed by TEM with the same resolution. Increment in the thickness of the sidewall of the functionalised MWNT after covalently immobilised enzyme confirmed the presence of enzyme. Similar observations of uniform coating of the *Candida rugosa* lipase to the MWNT via physically adsorption were also reported [20].

**Figure 4:**
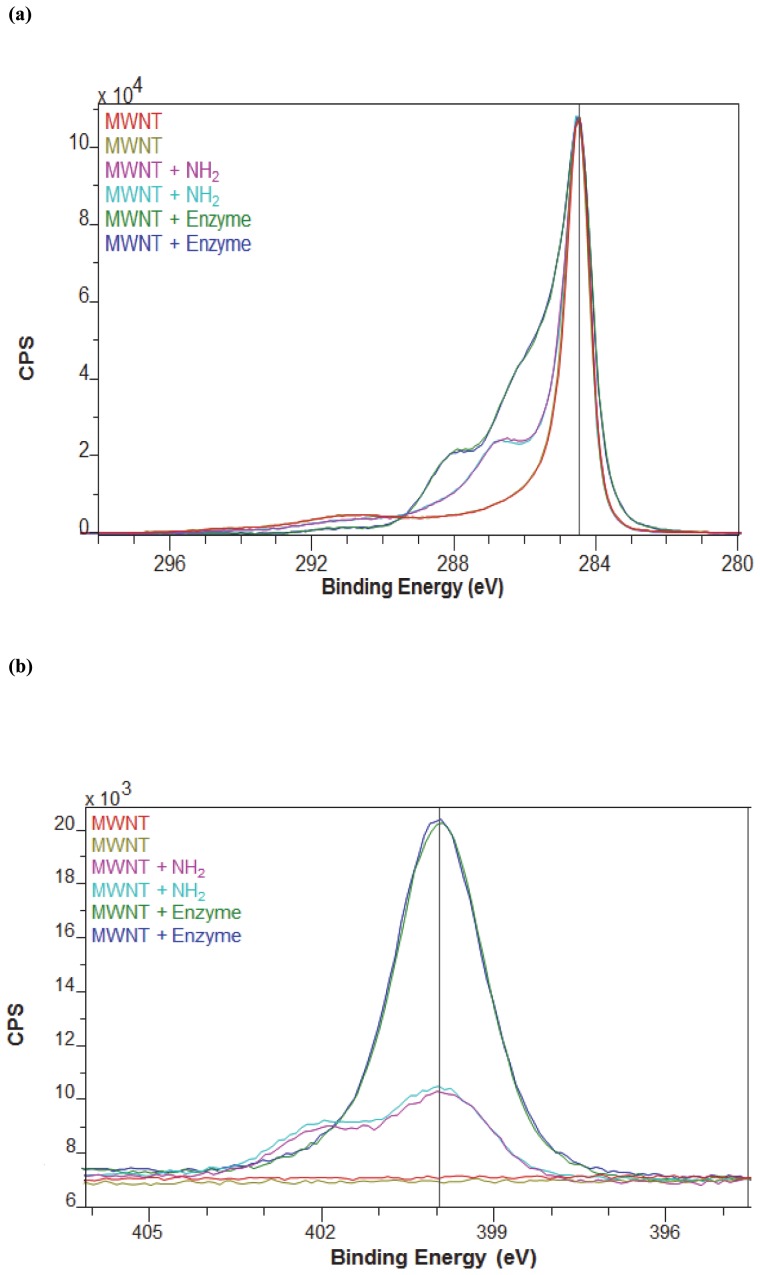
a) XPS spectrum of MWNT and MWNT bound lipase showing carbon 1 s high resolution spectra. b) XPS spectrum of MWNT and MWNT bound lipase showing nitrogen 1 s high resolution spectra.

XPS represents an effective technique for surface analysis [28] and was employed to determine the surface properties of the relative atomic composition of functionalised MWNT to immobilised lipase. High resolution XPS spectra for the as received MWNT, amino-functionalised MWNT and enzyme bound MWNT are shown in Figure 4a,b. Two points per sample were analysed and survey spectra were used to identify all elements present on the sample surfaces and to determine their concentrations. The high-resolution spectra were used to determine the presence or absence of chemically distinct species (e.g. functional groups). As shown in Figure 4a and b, there is a main peak at 284.6 eV for as-received MWNT, due to sp^2^ C-C bonds of graphitic carbon. Apart from a strong C-C peak, the additional peaks observed for amino-functionalised and enzyme bound MWNTs at higher binding energies are due to bonding of carbon atoms to other functional groups. For the amino-functionalised MWNTs, the additional binding energy at 286.9 eV is attributed to an amide group [29]. The absence of a peak around 288.0 eV also confirms the conversion of the carboxylic acid group to an amide. The binding energy at 401.0 eV in the N1s spectra is assigned to the formation of amides and amines on the carbon nanotubes, which further confirms the success of MWNT functionalisation as well as its covalent bonding to the enzyme. As expected, no peak was observed for the as received MWNT in the N1s spectrum. The atomic concentrations of oxygen and nitrogen in samples based on XPS analyses are presented in Table 1. The higher nitrogen content for the amino-functionalised MWNTs compared to as received nanotubes confirms the presence of amide groups on the surface of the nanotubes. The significant increase in both surface oxygen and nitrogen contents in nanotube bound enzymes indicates the covalent bonding between amino-functionalised MWNT and lipase (Table 1). Compared to that of MWNTs, the spectrum of lipase bound MWNTs shows an increase in the intensity of oxygen. This is attributed to the oxygen atoms in the immobilised lipase. A similar trend is also reported in a recent study of lipase immobilised on magnetic multi-walled carbon nanotubes [30].

**Table 1:** Atomic concentrations of MWNT, amino-functionalised MWNT and amino-functionalised MWNT bound enzyme from XPS experimental data.

Nanomaterial	Atomic concentration of oxygen (%)	Atomic concentration of nitrogen (%)
MWNT	1.0	–
Amino-functionalised MWNT	13	5
Amino-functionalised MWNT bound enzyme	20	14

All experiments in this study were carried out in triplicate with standard deviation below 5%.

**Figure 5:**
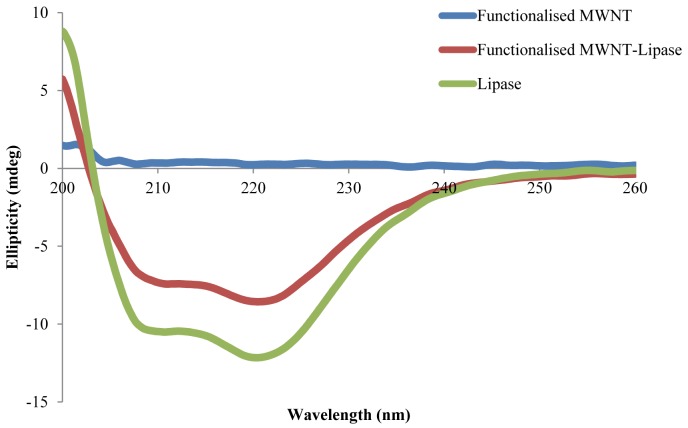
CD spectrum of free and MWNT bound enzyme.

The influence of covalent attachments on the secondary structure of enzyme was analysed by CD spectroscopy. The structural change of the immobilised lipase was compared to the free enzyme through CD spectra analysis (Fig. 5). MWNTs contribute to the spectrum of the nanotube bound lipase. The CD Pro software package (CONTIN) was used to interpret the secondary structure fractions. Compared to free enzyme, the amount of α-helix in MWNT-immobilised enzyme decreased, whereas β-sheet and unordered polypeptide amount increased (Table 2). Immobilised lipase retains approximately 80% of its native α- helix content, as obtained from the mean residue ellipticity at 222 nm. The degree of α-helix content of native lipase (63%) was retained after its immobilisation on magnetic multi-walled carbon nanotubes [29], with 62% retention of the α-helix content of the native lipase after immobilisation on multi-walled carbon nanotubes [31]. Recently, the α-helix content of the magnetic nanoparticle immobilised enzyme was not significantly changed and subsequently, the preserved secondary structure of the enzyme following nanoparticle conjugation demonstrated the high catalytic activity as compared to the native enzyme [32]. These results were encouraging where secondary structure changes remained unaffected contributing towards higher stability of the biocatalysts.

**Table 2:** Fractions of each type of secondary structure in lipase covalently immobilised to amino-functionalised MWNT.

Structure	H(r)	H(d)	S(r)	S(d)	Trn	Unrd
Native enzyme	0.607	0.265	0.002	0.002	0.009	0.116
Immobilised enzyme	0.491	0.189	0.006	0.013	0.095	0.206

Abbreviations: H(r), regular α-helix; H(d), distorted α-helix; S(r), regular β sheet; s(d), distorted β sheet; Trn, Turn; Unrd, unordered. All experiments in this study were carried out in triplicate with standard deviation below 5%.

### Effects of pH and Temperature on the Catalytic Activity of Free and Bound Biocatalyst

**Figure 6:**
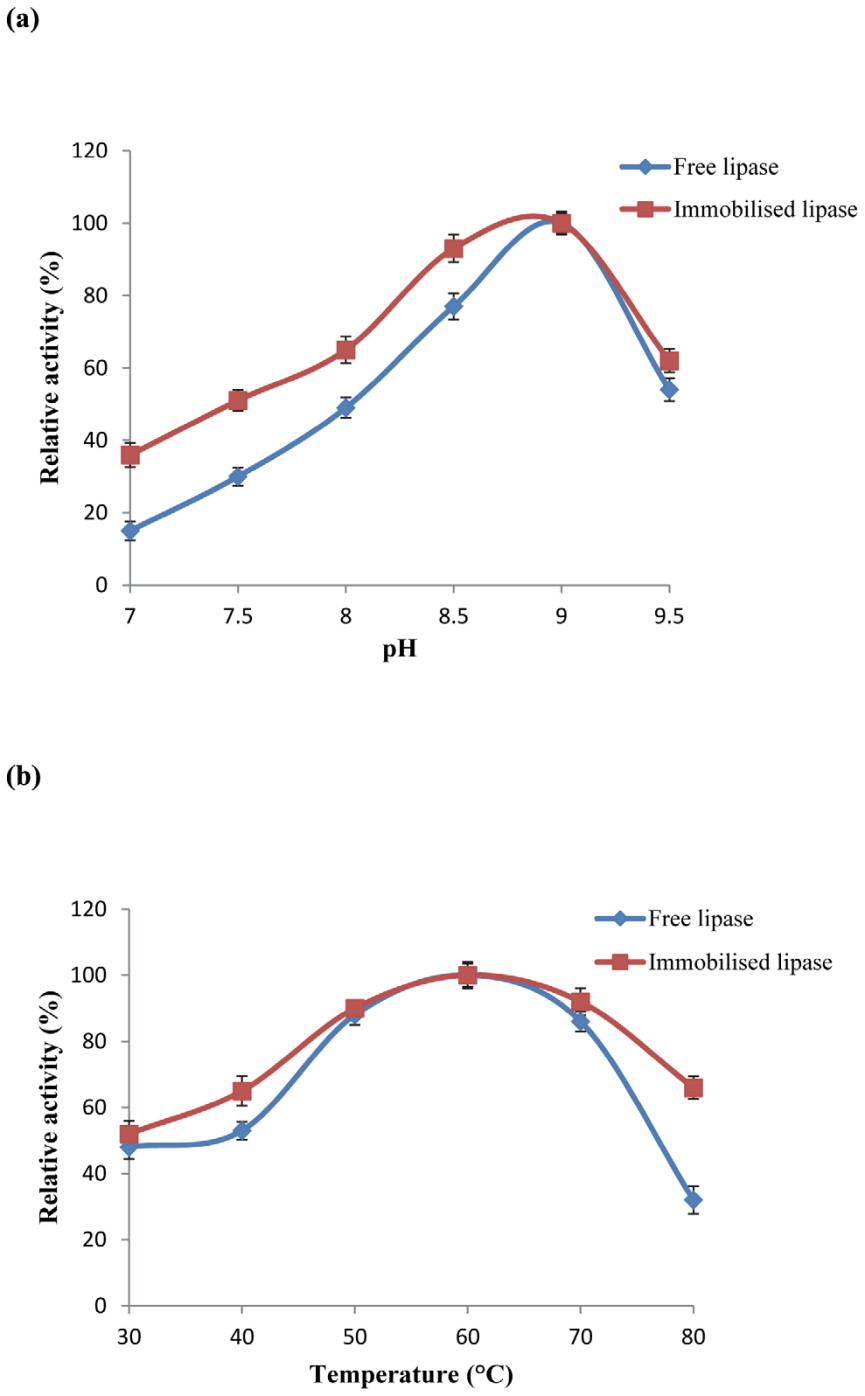
a) Effect of pH on the activity of free and immobilised lipase. Footnote: The lipolytic activity was determined following the method of Winkler and Stuckmann (1979) [21], using p-NP palmitate as substrate. Bars indicate the standard deviation from triplicate determinations. b) Effect of temperature on the activity of free and immobilised lipase. Footnote: The lipolytic activity was determined following the method of Winkler and Stuckmann [21], using p-NP palmitate as substrate. Bars indicate the standard deviation from triplicate determinations.

The optimum pH value for the activity of both free and immobilised enzymes was found to be 9.0 (Fig. 6a). The immobilised enzyme was comparatively more active at lower pH values as compared to high pH value. This result is consistent with the previously reported study where cyanogen bromide (CNBr) activated octyl-sepharose support was used for immobilising *Thermomyces lanuginosus* lipase at pH 9.0 [33].

### Kinetic Parameters

Kinetic parameters of free and immobilised lipase were determined by measuring initial reaction rates for each form with varying amounts of substrates (p-NP palmitate). For all forms, Michaelis-Menten kinetic behaviour was observed. As shown in Table 3, *K*_M_ value of the immobilized lipase (4.5 mM) was slightly more than that of the free enzyme (3.1 mM). The maximum reaction rates (*Vmax*) of the free and immobilised enzyme were 20.1 U/mg and 22.9 U/mg. A 4-fold increase in the *K*_M_ value was observed for the immobilised enzyme compared to free enzyme [36]. *K*_M_ value of the immobilized *Thermomyces lanuginosus* lipase on hydrophobic and hydrophilic mesoporous supports was 3.4 and 1.5 mM respectively, compared to a free enzyme value of 0.8 mM. These changes in the kinetic parameters indicate that covalent binding of lipase onto the glutaraldehyde-activated functionalised nanotubes resulted in change of affinity for the substrate. This may be due to slightly decreased access of the substrate to the active site of enzyme in the bound lipase [37].

**Table 3:** Kinetics of free and immobilised lipase using p-NP palmitate as substrate.

Parameters	Free enzyme	Immobilised enzyme
*K_M_* (mM)	3.1	4.5
*Vmax* (U/mg)	20.1	22.9

Footnote: Initial rates were estimated from the extent of substrate hydrolysis during 10 min incubation at 60°C. Reaction mixtures contained 10–60 mM p-NP palmitate, free and immobilised lipase (2 U), in 2.9 mL in 50 mM Tris buffer (pH 9.0). Kinetic parameters were calculated from GraphPad software (version 6.0). All experiments in this study was carried out in triplicate with standard deviation below 5%.

### Thermal Stability of Free and Immobilised Lipase

**Figure 7:**
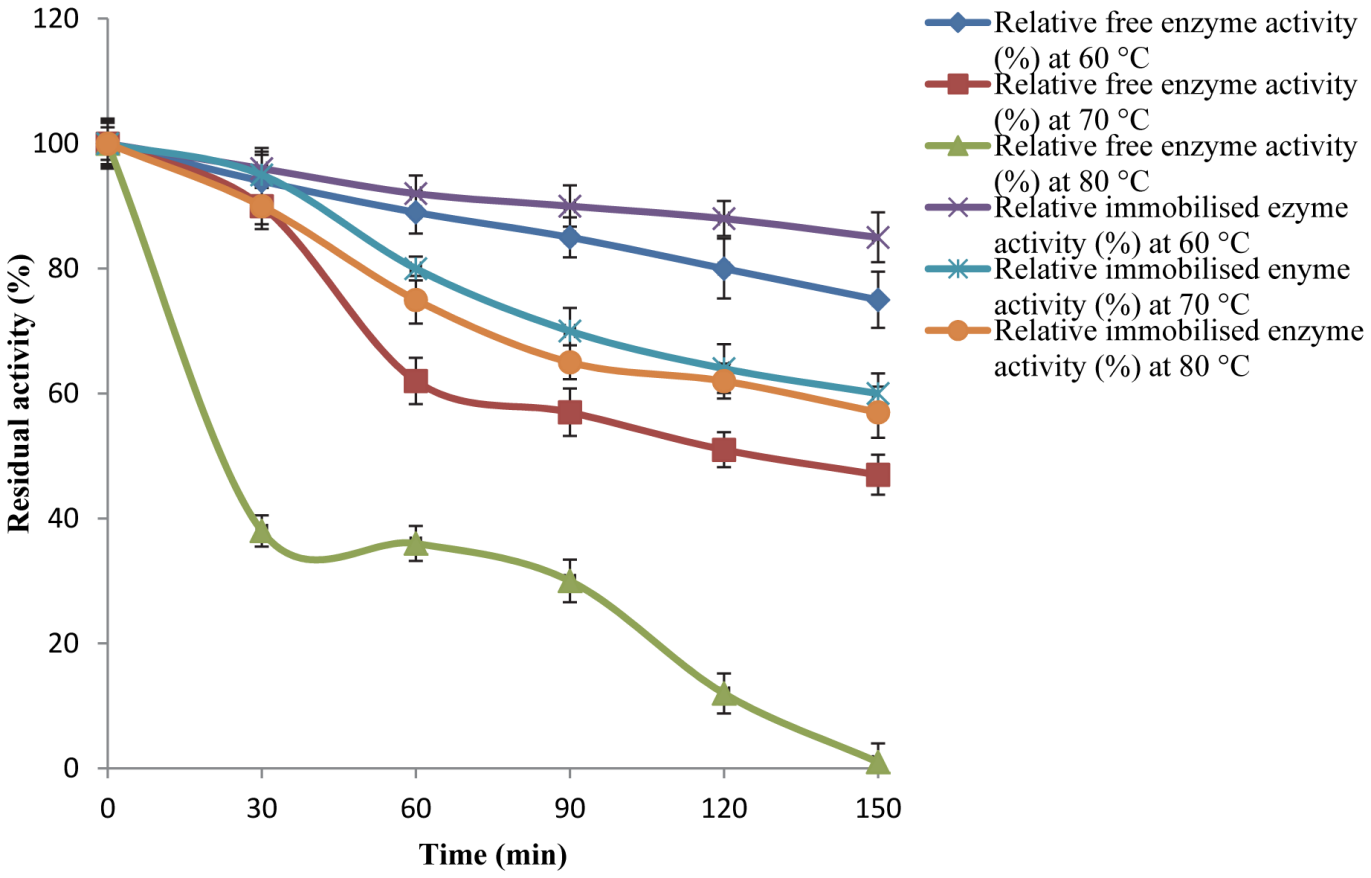
Thermostability studies of free and the immobilised lipase. Footnote: The lipolytic activity was determined following the method of Winkler and Stuckmann [21], using p-NP palmitate as substrate. Bars indicate the standard deviation from triplicate determinations.

Figure 7 shows the thermostability of free and immobilised lipases at temperatures of 60, 70 and 80°C, respectively. It is evident that the thermal stability of the lipase immobilised on nanotubes was improved and prominent compared to the free enzyme at 80°C. The free enzyme lost its activity after 2 h of incubation at 80°C, whereas the immobilised lipase retained 57% of its initial activity after 2 h incubation (Fig. 7). This trend is in agreement with the findings reported in the literature. For example, all activity was lost in *Thermomyces lanuginosus* lipase dissolved in phosphate buffer at 80°C after 2 h and authors claimed the improvement in thermostability of the enzyme using an ionic liquid [38]. The increased stability observed for the immobilised enzyme, either thermal or due to interactions with solvents, observed may be attributed to a reduction in the protein structure mobility, due to anchorage to the support promoted by the covalent bonds and subsequent translation of the rigidity at each anchorage point to the whole enzyme structure, thus shielding it from the denaturing effects of the environment [39].

In a recent study, the enzyme-carbon nanotube interactions significantly improved the thermal stability and catalytic activity of enzymes and confirmed that a significant confirmation is controlled by the functional group on the nanomaterial’s surface and less on their geometry [19]. Thus, effect of functionalisation agent (ethylenediamine) in conjugation with coupling agent (HATU) to MWNTs, played an important role in improving the reaction yield of amino-functionalisation and subsequently enhanced the catalytic property of immobilised enzyme in this present study. These nanoscale carriers are stable at high temperatures and high enzyme-loadings on MWNT can be achieved [31].

### Biocatalyst Reusability Study

**Figure 8:**
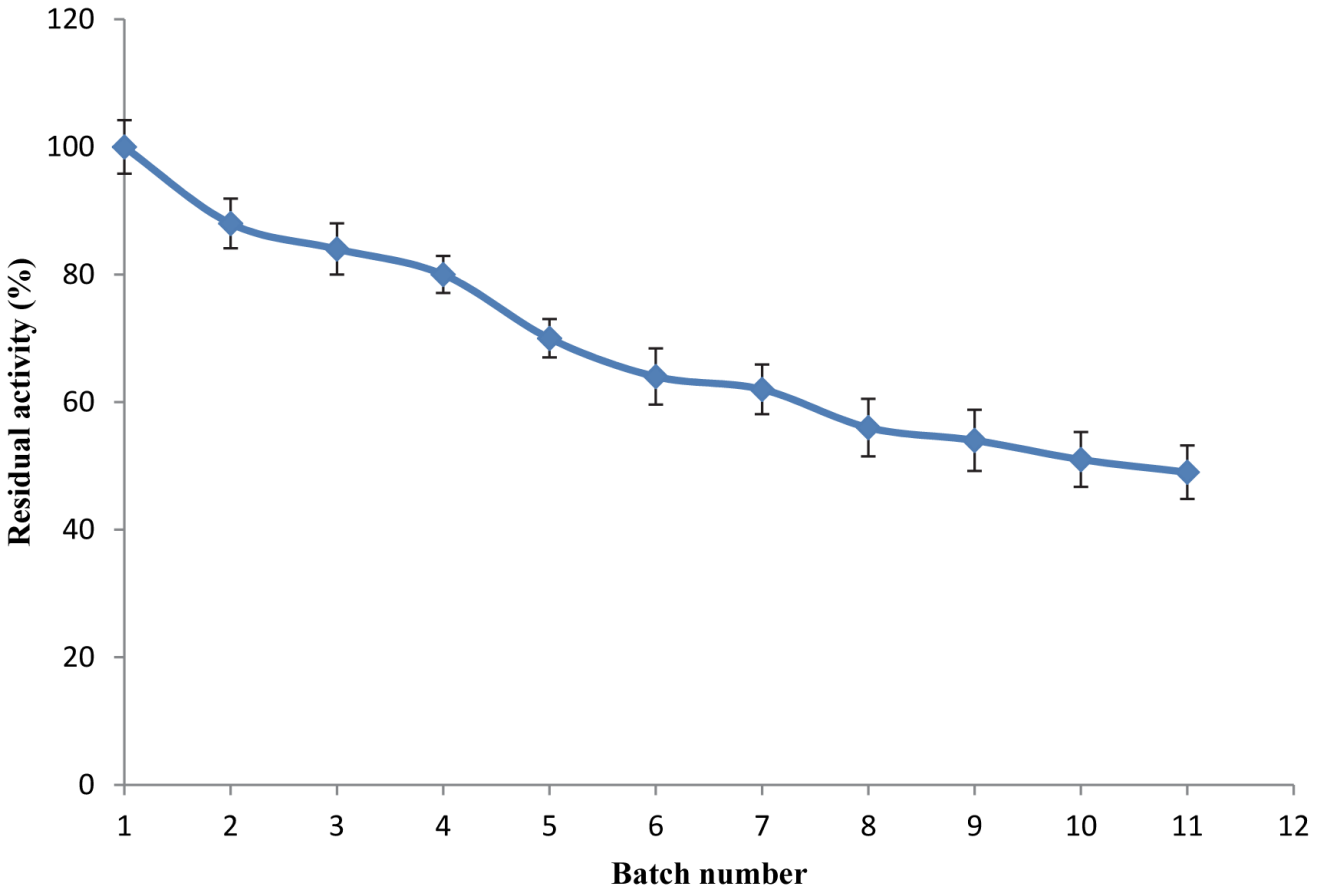
Reusability study of the immobilised lipase. Footnote: The lipolytic activity was determined following the method of Winkler and Stuckmann [21], using p-NP palmitate as substrate. Bars indicate the standard deviation from triplicate determinations. Residual activity of immobilised enzyme at different cycles. 1 cycle = 100±4.2; 2 cycle = 88±3.9; 3 cycle = 84±4.0; 4 cycle = 80±2.9; 5 cycle = 70±3.0; 6 cycle = 64±4.4; 7 cycle = 62±3.9; 8 cycle = 56±4.5; 9 cycle = 54±4.8; 10 cycle = 51±4.3; 11 cycle = 49±4.2.

The immobilised enzyme retained residual activity above 80% up to the 4^th^ recycle, demonstrating stability up to 10 cycles of 10 min at 60°C, and retaining approximately half of its initial activity at the end the enzymatic derivative (Fig. 8). Pavlidis et al. [19] demonstrated that the lipase (Cal B) activity of the immobilised enzyme in amine-functionalised carbon nanotube preparations retain more than 50% of their activity after five repeated uses at 60°C.This immobilisation method reduces the enzyme leaching into media and decreases protein contamination in the product. Superior reusability of lipase was observed due to nanotube binding, thus presenting a cost-based argument for its potential role in economically viable enzyme catalysed process.

## Conclusion

Successful functionalisation and post immobilisation changes in the enzyme were confirmed at structure-function level using analytical techniques such as TEM and spectroscopy (XPS, CD, FTIR), respectively. The kinetics of immobilised enzyme indicated that the enzyme undergoes a slight change in kinetics parameters during immobilisation, while retaining the same pH and temperature optima of the free lipase. The satisfactory immobilisation yield, improved thermostability, and encouraging reusability of nanotube bound lipase, makes this nanobiocatalyst system of potential interest for biosensor applications.

## Supporting Information

Figure S1 **Schematic representation of the covalent immobilisation of enzyme to amino-functionalised MWNT.**
(TIF)

## References

[1] Kim J, Grate JW, Wang P (2008) Nanobiocatalysis and its potential applications. Trends Biotechnol 26: 639–646. 18804884 10.1016/j.tibtech.2008.07.009

[2] Ansari SA, Husain Q (2012) Potential applications of enzymes immobilized on/in nanomaterials: a review. Biotechnol Adv 30: 512–523. 21963605 10.1016/j.biotechadv.2011.09.005

[3] Puri M, Barrow CJ, Verma ML (2013) Enzyme immobilization on nanomaterials for biofuel production. Trends Biotechnol 31: 215–216. 23410582 10.1016/j.tibtech.2013.01.002

[4] Verma ML, Barrow CJ, Puri M (2013) Nanobiotechnology as a novel paradigm for enzyme immobilisation and stabilisation with potential applications in biodiesel production. Appl Microbiol Biotechnol 97: 23–39. 23132346 10.1007/s00253-012-4535-9

[5] Asuri P, Bale SS, Pangule RC, Shah DA, Kane RS, et al (2007) Structure, function, and stability of enzymes covalently attached to single-walled carbon nanotubes. Langmuir 23: 12318–12321. 17944500 10.1021/la702091c

[6] Shi Q, Yang D, Su Y, Li J, Jiang Z, et al (2007) Covalent functionalisation of multi-walled carbon nanotubes by lipase. J Nanopart Res 9: 1205–1210.

[7] Lee SH, Doan TTN, Won K, Ha SH, Koo YM (2010) Immobilisation of lipase within carbon nanotube-silica composites for non-aqueous reaction systems. J Mol Catal B Enzym 62: 169–172.

[8] Johnson PA, Park HJ, Driscoll AJ (2011) Enzyme nanoparticle fabrication: magnetic nanoparticle synthesis and enzyme immobilisation. Methods Mol Biol 679: 183–191. 20865397 10.1007/978-1-60761-895-9_15

[9] Shim M, Kam NWS, Chen RJ, Li Y, Dai H (2002) Functionalisation of carbon nanotubes for biocompatibility and biomolecular recognition. Nano Lett 2: 285–288.

[10] Pavlidis IV, Tsoufis T, Enotiadis A, Gournis D, Stamatis H (2010) Functionalized multi-wall carbon nanotubes for lipase immobilisation. Adv Eng Mater 12: B179–B183.

[11] Cruz JC, Pfromm PH, Tomich JM, Rezac ME (2010) Conformational changes and catalytic competency of hydrolases adsorbing on fumed silica nanoparticles: I. Tertiary structure. Colloid Surf B 79: 97–104. 10.1016/j.colsurfb.2010.03.036 20434319

[12] Verma ML, Kanwar SS (2008) Properties and application of poly (methacrylic acid-co-dodecyl methacrylate-*cl*-*N,N*-methylene bisacrylamide) hydrogel immobilized *Bacillus cereus* MTCC 8372 lipase for the synthesis of geranyl acetate. J Appl Polym Sci 110: 837–846.

[13] Verma ML, Chaudhary R, Tsuzuki T, Barrow CJ, Puri M (2013) Immobilization of β-glucosidase on a magnetic nanoparticle improves thermostability: application in cellobiose hydrolysis. Bioresour Technol 135: 2–6. 23419989 10.1016/j.biortech.2013.01.047

[14] Verma ML, Rajkhowa R, Wang X, Barrow CJ, Puri M (2013) Exploring novel ultrafine Eri silk bioscaffold for enzyme stabilisation in cellobiose hydrolysis. Bioresour Technol, doi: 10.1016/j.biortech.2013.01.065. 10.1016/j.biortech.2013.01.065 23462595

[15] Chronopoulou L, Kamel G, Sparago C, Bordi F, Lupi S, et al (2011) Structure-activity relationships of *Candida rugosa* lipase immobilised on polylactic acid nanoparticles. Soft Matter 7: 2653–2662.

[16] Puri M, Gupta S, Kaur A, Pahuja A, Kennedy JF (2010) Cell disruption and covalent immobilisation of b-galactosidase from *Kluyveromyces marxianus* YW-1 for lactose Hydrolysis. App Biochem Biotech 160: 98–108. 10.1007/s12010-009-8542-y 19198767

[17] Puri M, Kaur S, Kennedy JFK (2005) Covalent immobilization of naringinase for the transformation of flavonoids. J Chemical Technol Biotechnol 80: 1160–1165.

[18] Verma ML, Barrow CJ, Kennedy JF, Puri M (2012) Immobilization of β-d-galactosidase from *Kluyveromyces lactis* on functionalized silicon dioxide nanoparticles: characterization and lactose hydrolysis. Int J Biol Macromol 50: 432–437. 22230612 10.1016/j.ijbiomac.2011.12.029

[19] Pavlidis IV, Vorhaben T, Tsoufis T, Rudolf P, Bornscheuer UT, et al (2012) Development of effective nanobiocatalytic systems through the immobilisation of hydrolases on functionalized carbon-based nanomaterials. Bioresour Technol 115: 164–171. 22113071 10.1016/j.biortech.2011.11.007

[20] Shah S, Solanki K, Gupta MN (2007) Enhancement of lipase activity in non-aqueous media upon immobilization on multi-walled carbon nanotubes. Chem Central J 1: 30. 10.1186/1752-153X-1-30 PMC2211749 18047656

[21] Boncel S, Zniszczol A, Szymanska K, Mrowiec-Bialon J, Jarzebski A, et al. (2013) Alkaline lipase from *Pseudomonas florescens* non-covalently immobilised on pristine versus oxidised multi-walled carbon nanotubes as efficient and recyclable systems in the synthesis of Solketal esters. Enzyme Microb Technol doi:10.1016/j.enzmictec.2013.05.003. 10.1016/j.enzmictec.2013.05.003 23931692

[22] Raghavendra T, Basak A, Manocha LM, Shah AR, Madamwar D (2013) Robust nanobioconjugates of *Candida antartica* lipase B-multiwalled carbon nanotubes: characterization and application for multiple usages in non-aqueous biocatalysis. Bioresour Technol 140: 103–110. 23685646 10.1016/j.biortech.2013.04.071

[23] Bradford MM (1976) A rapid and sensitive method for the quantitation of microgram quantities of protein utilizing the principle of protein-dye binding. Anal Biochem 72: 248–254. 942051 10.1016/0003-2697(76)90527-3

[24] Winkler UK, Stuckmann M (1979) Glucogen hyaluronate and some other polysaccharides greatly enhance the formation of exolipase by *Serratia marcescens.* . J Bacteriol 138: 663–670. 222724 10.1128/jb.138.3.663-670.1979 PMC218088

[25] Ganesan A, Moore BD, Kelly SM, Price NC, Rolinski OJ, et al (2009) Optical spectroscopic methods for probing the conformational stability of immobilised enzymes. Chemphyschem 10: 1492–1499. 19360797 10.1002/cphc.200800759

[26] Wang S, Liang Z, Liu T, Wang B, Zhang C (2006) Effective amino functionalization of carbon-nanotubes for reinforcing epoxy polymer composites. Nanotechnology 17: 1551–1557. 26558557 10.1088/0957-4484/17/6/003

[27] Ramanathan T, Fisher FT, Ruoff RS, Brinson LC (2005) Amino-functionalized carbon nanotubes for binding to polymers and biological systems. Chem Mater 17: 1290–1295.

[28] Liu Y, Yu ZL, Zhang YM, Guo DS, Liu YP (2008) Supramolecular architectures of β-cyclodextrin-modified chitosan and pyrene derivatives mediated by carbon nanotubes and their DNA condensation. J Am Chem Soc 130: 10431–10439. 18627155 10.1021/ja802465g

[29] Eda G, Fanchini G, Chhowalla M (2008) Large-area ultrathin films of reduced grapheme oxide as a transparent and flexible electronic material. Nature Nanotechnol 3: 270–274. 18654522 10.1038/nnano.2008.83

[30] Tan H, Feng W, Ji P (2012) Lipase immobilized on magnetic multi-walled carbon nanotubes. Bioresour Technol 115: 172–176. 22115533 10.1016/j.biortech.2011.10.066

[31] Ji P, Tan HS, Xu X, Feng W (2010) Lipase covalently attached to multiwalled carbon nanotubes as an efficient catalyst in organic solvent. Aiche J 56: 3005–3011.

[32] Talbert JN, Goddard JM (2013) Characterization of lactase-conjugated magnetic nanoparticles. Process Biochem 48: 656–662.

[33] Rodrigues RC, Godoy CA, Volpato G, Ayub MAZ, Fernandez-Lafuente R, et al (2009) Immobilization-stabilization of the lipase from *Thermomyces lanuginosus*: critical role of chemical amination. Process Biochem 44: 963–968.

[34] Ondul E, Dizge N, Albayrak N (2012) Immobilization of *Candida antartica* A and *Thermomyces lanuginosus* lipases on cotton terry cloth fibrils using polyethyleneimine. Colloid Surf B Biointer 95: 109–114. 10.1016/j.colsurfb.2012.02.020 22421414

[35] Fernandez-Lafuente R (2010) Lipase from *Thermomyces lanuginosus*: uses and prospects as an industrial biocatalyst. J Mol Catal B Enzym 62: 197–213.

[36] Sorensen MH, Ng JBS, Bergstrom L, Alberius PCA (2010) Improved enzymative activity of *Thermomyces lanuginosus* lipase immobilized in a hydrophilic particulate mesoporous carrier. J Colloid Interface Sci 343: 359–365. 20022021 10.1016/j.jcis.2009.11.014

[37] Tu M, Zhang X, Kurabi A, Gilkes N, Mabee W, et al (2006) Immobilization of β-glucosidase on Eupergit C for lignocellulose hydrolysis. Biotechnol Lett 28: 151–156. 16489491 10.1007/s10529-005-5328-3

[38] Akanbi TO, Barrow CJ, Byrne N (2012) Increased hydrolysis by *Thermomyces lanuginosus* lipase for omega-3 fatty acids in the presence of a protic liquid. Catal Sci Technol 2: 1839–1841.

[39] Taqieddin E, Amiji M (2004) Enzyme immobilization in novel alginate-chitosan core-shell microcapsules. Biomaterials 25: 1937–1945. 14738858 10.1016/j.biomaterials.2003.08.034

