# Supplementary figures and images for: Enzyme Immobilisation on Amino-Functionalised Multi-Walled Carbon Nanotubes: Structural and Biocatalytic Characterisation
Source: PLoS One. 2013 Sep 12;8(9):e73642. doi: 10.1371/journal.pone.0073642 (PMC3772012; doi:10.1371/journal.pone.0073642)

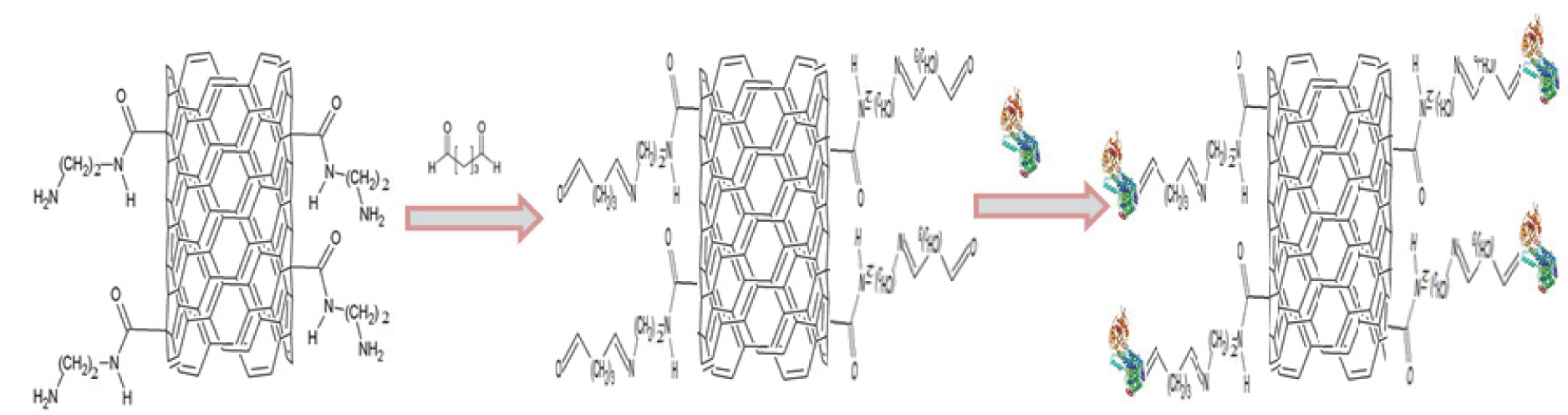

Supplement: Figure S1 — Schematic representation of the covalent immobilisation of enzyme to amino-functionalised MWNT. (TIF) [file pone.0073642.s001.tif]
